# Dynamics of marine bacterial community diversity of the coastal waters of the reefs, inlets, and wastewater outfalls of southeast Florida

**DOI:** 10.1002/mbo3.245

**Published:** 2015-03-05

**Authors:** Alexandra M Campbell, Jay Fleisher, Christopher Sinigalliano, James R White, Jose V Lopez

**Affiliations:** 1 Center of Excellence in Coral Reef Ecosystem Research, Nova Southeastern UniversityDania Beach, Florida, 33004; 2 School of Osteopathic Medicine, Nova Southeastern University3301 College Avenue, Davie, Florida, 33004; 3 Atlantic Oceanographic and Meteorological Laboratory, National Oceanic and Atmospheric AdministrationMiami, Florida, 33149; 4 Resphera BiosciencesBaltimore, Maryland, 21231

**Keywords:** 16S rRNA, bacterioplankton, coral reef, South Florida

## Abstract

Coastal waters adjacent to populated southeast Florida possess different habitats (reefs, oceanic inlets, sewage outfalls) that may affect the composition of their inherent microbiomes. To determine variation according to site, season, and depth, over the course of 1 year, we characterized the bacterioplankton communities within 38 nearshore seawater samples derived from the Florida Area Coastal Environment (FACE) water quality survey. Six distinct coastal locales were profiled – the Port Everglades and Hillsboro Inlets, Hollywood and Broward wastewater outfalls, and associated reef sites using culture-independent, high-throughput pyrosequencing of the 16S rRNA V4 region. More than 227,000 sequences helped describe longitudinal taxonomic profiles of marine bacteria and archaea. There were 4447 unique operational taxonomic units (OTUs) identified with a mean OTU count of 5986 OTUs across all sites. Bacterial taxa varied significantly by season and by site using weighted and unweighted Unifrac, but depth was only supported by weighted Unifrac, suggesting a change due to presence/absence of certain OTUs. Abundant microbial taxa across all samples included *Synechococcus*, Pelagibacteraceae, Bacteroidetes, and various Proteobacteria. Unifrac analysis confirmed significant differences at inlet sites relative to reef and outfalls. Inlet-based bacterioplankton significantly differed in greater abundances of Rhodobacteraceae and Cryomorphaceae, and depletion of SAR406 sequences. This study also found higher counts of Firmicutes, Chloroflexi, and wastewater associated SBR1093 bacteria at the outfall and reef sites compared to inlet sites. This study profiles local bacterioplankton populations in a much broader context, beyond culturing and quantitative PCR, and expands upon the work completed by the National Oceanic and Atmospheric Administration FACE program.

## Introduction

Bacteria and archaea participate in the biogeochemical cycling of nutrients and organic material (Azam et al. 1983; DeLong 2009), atmospheric gas production, and form a large portion of organismal and genetic diversity in the oceans. For example, the continental shelf habitats host about 5 × 10^5^ cells/mL of seawater (Whitman et al. 1998). *Prochlorococcus* and other bacterioplankton appear to contribute the most diversity to oceanic waters, and the diversity of these communities can be influenced by environmental conditions. Seasonal, diel, and spatial differences, often over short distances, have strong effects on microbial community structures (Gifford et al. 2014). Alternations between wet and dry weather and temperature change the nutrient levels and alter freshwater input from terrestrial ecosystems, which affects phytoplankton abundance and indirectly changes microbial structure (McArthur 2006). Depth is also an important factor to consider. Field et al. (1997) found that SAR11 bacteria show depth-specific distribution.

As one example for seasonal effects on microbial diversity, Gilbert et al. (2009, 2010a) demonstrated that microbial communities, using 16S rRNA, varied temporally with seasons in the Western English Channel, a region where warm temperate and cold temperate waters converge (Southward et al. 2005). The highest microbial diversity occurred in the winter months, although temperatures between the winter (January–April) and the summer (August) months ranged over 5–6°C. Chlorophyll-a concentrations also varied greatly from winter to summer, with the highest levels occurring in the summer. The apparent bloom of photosynthetic microorganisms, including *Synechococcus*, resulted in lower microbial diversity (Gilbert et al. 2010a). Similarly, a recent study by Gifford et al. (2014) showed variable bacterial populations likely driven by their ability to process available compounds when specific nutrients and primary producer presence fluctuated according to season.

In contrast to the Western English Channel, southeast Florida is a region where tropical and temperate waters converge (Banks et al. 2008). This region also encompasses several large ecosystems centered on water dynamics, such as the Everglades swamp, mangrove estuaries, and a coral reef tract, the largest in the United States. The humid tropical savannah climate, attracting much of the population to the region, is based on the Köppen climate scale (Peel et al. 2007), and is distinguished by a distinct wet (May–September) and dry season (Banks et al. 2008).

The coastline of southeast Florida, comprised Miami-Dade, Broward, and Palm Beach counties, extends 142 km, and is continuously developed and urbanized with a dense population of 5,710,953 people (U.S. Census 2010, http://quickfacts.census.gov/qfd/states/12000.html). To deal with the increased drainage demand from the expanding population, the Army Corps of Engineers built drainage canals causing a change in surface and ground water flows (Sklar et al. 2001). These drainage canals remain a major carrier of agricultural and urban runoff (SFWMD 2010). About 57% of southeast Florida's population between these counties relies on centralized sewers, with the remaining 40% dependent on in-ground (relatively untreated) wastewater disposal (Futch et al. 2011). Treated wastewater is removed by a series of outfalls, two of which are located in Broward County, which discharge directly into the coastal ecosystem. An extensive system of coastal canals likely carry pollutants into the marine environment such as storm water, urban and agricultural runoff, and reckless waste dumping (Futch et al. 2011). The drainage canals connect to the Intracoastal Waterway (ICW), which spans from Fernandina Harbor to Miami Harbor and requires periodic dredging. It connects to the Atlantic Ocean via a series of inlets, which are noted as major pollution sources (Puglise and Kelty 2007; Lapointe and Bedford 2010). In addition, five open ocean-treated wastewater outfalls operate in the southeast Florida region, located off Miami-Dade, Broward, and Palm Beach Counties. All of the outfall sites have terminations at water depths of about 28 m, near the westerly boundary of the Florida Current, and at or beyond the outer reef line (for bathymetry see Banks et al. 2008; Walker et al. 2008; Walker 2012). Effluent from the outfalls rises and eventually mixes with surrounding water as it rises to the surface. The amount flowing through the outfalls can vary considerably from day to day, and is strongly dependent on the local rainfall. The number of outfalls has decreased from 10 outfalls operating in 1972 to 6 at the time of writing. A summary of outfall characteristics can be found in Koopman et al. (2006), and in the 2010 annual report of the Florida Department of Environmental Protection (FDEP 2010).

Biogeographically, the southeast Florida coast supports a high diversity of macro- and microorganisms (Robertson and Cramer 2014). Much of this diversity stems from the local coral reef ecosystem (Banks et al. 2008; Walker et al. 2008, 2012). The Florida reef tract begins in southern Martin County and extends through Broward and Miami-Dade counties before entering the Florida Keys reef tract, a distance of about 125 km (Banks et al. 2008). The reefs run parallel to the shoreline, are dominated by massive stony corals such as *Orbicella* spp., and form a reef complex composed of an inner, middle, and outer reef system (Banks et al. 2008). More studies continue to characterize these reefs, which exhibit many of the hallmarks of a reef in decline and under stress (Wilkinson 2008; Gilliam 2010; Walker et al. 2012; Jackson et al. 2014). Coral reefs represent one of the most biologically diverse habitats on the planet, and their microbial composition remains under intensive investigation (Knowlton and Rohwer 2003; Negandhi et al. 2010; White et al. 2012; Webster et al. 2013). However, increases in the amount of rainfall correlate with the number of fecal indicator bacteria (FIB), used to gauge fecal contamination in water (Brownwell et al. 2007; Yeo et al. 2013) and which can impact reef health. Previous work on FIB in southeast Florida waters and beaches have shown seasonal spikes of specific taxa due to nonpoint source contamination sewage (Shibata et al. 2004), storm water (Shibata et al. 2004; Brownwell et al. 2007), sand resuspension (Hartz et al. 2008), dog feces (Wright et al. 2009), and human shedding (Abdelzaher et al. 2010) of commensal organisms such as *Enterococci* and *Staphylococcus aureus* (Elmir et al. 2007). Inputs of sewage-related bacteria have been implicated as a cause of disease such as white pox disease in *Acropora palmata* (a coral) caused by *Serratia marcescens* (a bacterium) (Patterson et al. 2002), potentially stemming from a variety of sources including human sewage (Sutherland et al. 2010).

Because the majority of microbial diversity is unculturable, culture-independent molecular analyses have become commonplace and contribute a more detailed view of microbial assemblages (known as “microbiomes”), including their potential function (Handelsman 2004; DeLong 2005, 2009; Jiménez et al. 2014; Kinross et al. 2014; Tuohy and Scott 2014). Early microbiome projects, such as the Sargasso Sea sequencing project (Venter et al. 2004) and its larger offshoot the Global Ocean Sampling Expedition (Rusch et al. 2007), have expanded knowledge of the open ocean by finding novel phylotypes and functional genes (Venter et al. 2004; Rusch et al. 2007; Temperton and Giovannoni 2012). As sequencing power has increased, other studies such as the Human Microbiome Project (Human Microbiome Consortium 2012), and the Earth Microbiome Project (Gilbert et al. 2010b) and our laboratory's sponge microbiome profiles (White et al. 2012; Cuvelier et al. 2015), have described microbial diversity patterns across diverse hosts and habitats, revealing a nonrandom distribution of microbial taxa across space or time (Fuhrman 2009; Steele et al. 2011; Zinger et al. 2011; Shogan et al. 2013).

Data generated from this study expand upon the previous work performed by the National Oceanic and Atmospheric Administration (NOAA) Florida Area Coastal Environment (FACE) water quality program (http://www.aoml.noaa.gov/themes/CoastalRegional/projects/FACE/faceweb.htm). FACE goals were to understand the various processes that shape the coastal environment and the relationship of public health to environmental health by identifying key nutrients, and microbial contaminants in the coastal environment, namely through culturing and quantitative PCR. The capabilities of DNA pyrosequencing were utilized in this study to complement data generated by the NOAA FACE program, and will allow the addressing of new objectives: (a) a comparison of bacterioplankton taxonomy across sites, (b) characterizing variation in microbial communities with site type (reef vs. outfall vs. inlet), (c) determining any differences in microbial communities due to seasonality (wet vs. dry), and (d) assessing any differences in microbial composition with depth.

## Experimental Procedures

### Seawater sample collection

Bacterioplankton samples characterized in this study were collected as part of a broader regional water quality study that also investigated current profiles, nutrient concentrations, viable fecal indicator concentrations, and molecular microbial host-source tracking of fecal indicators by quantitative PCR conducted by the FACE program of the NOAA Atlantic Oceanographic and Meteorological Laboratory (AOML). We report here only the results of the high-throughput sequencing, metataxonomic, and bioinformatic analyses of a subset of samples from this broader water quality study. The Doppler current profiles, physical oceanographic data, nutrient concentrations, and other microbiological data from the broader regional study are reported elsewhere.

All sampling for this study took place from the NOAA ship R/V Hildebrand with a Seabird SBE 19V2 CTD (conductivity–temperature–density measurement sensor suite) holding six 2 L bottles. The CTD sensor package measured depth, temperature, conductivity, pH, redox potential, dissolved oxygen, chlorophyll-a, and turbidity as per CTD manufacturer's instructions. In addition to these in situ physical measurements, a variety of chemical/nutrient measurements were conducted on water samples returned to the laboratory, including chlorophyll-a, phaeopigments, turbidity, oxygen saturation, total suspended solids (TSS), pH, oxygen reduction potential, total nitrogen, nitrates, ammonium, phosphorus, and silica (Table S1). Water samples for these laboratory-based chemical/nutrient water quality parameters were collected and analyzed as described previously (Carsey et al. 2011).

Seawater samples were collected bimonthly at the Broward (adjacent to the Hillsboro Inlet in Pompano Beach, FL) and Hollywood outfalls, reef tract, and coastal inlet sites shown in Table1 and Figure S1 from varying depths (surface, mid, and bottom) and seasons totaling 38 samples. Sample collection at the outfalls was determined by the location of the surface boil. If there were strong currents, the surface boil would be further from the pipe and could be missed (J. Stamates, pers. comm.). Overall, samples were collected from water <9.14 m. Only the Broward samples had the mid-depth sampled for metataxonomic analysis. Samples were collected between high and low tides around the Port Everglades (Table S2) and North Broward Outfalls (Table S3) using clean plastic 2L Niskin sampling bottles (attached to the CTD rosette described earlier), which close at a computer-specified depth. Replicate seawater samples were collected from the Niskin bottles aboard ship into sterile 1-L polypropylene bottles and kept on ice until return to the laboratory. For each sample, 1 L of seawater was filtered for genomic characterization using sterile cellulose nitrate (47 mm diameter, 0.45 micron pore size) membrane filters (Whatman, Little Chalfont, United Kingdom) via vacuum filtration. The filters were then folded with flame sterilized forceps and placed into sterile “lysing matrix A” bead beat tubes (MPBiomedicals, Inc., Santa Ana, CA), and stored frozen at −80°C for later processing. Then, total community genomic DNA was extracted from each filter with the FastDNA™ Spin Kit (MPBiomedicals, Inc.), according to the manufacturer's instructions as described previously (Sinigalliano et al. 2010). Purified DNA extracts were stored frozen at −80°C until analyzed.

**Table 1:** Overview of bacterioplankton collection locations and site types

Site ID	Latitude	Longitude	Habitat type
BR7	26.2039	−80.0683	Reef track
BR10	26.254	−80.0621	Outfall
BR14	26.2618	−80.0855	Coastal area, inlet
HW4	26.0163	−80.087	Outfall
HW9	26.0673	−80.0851	Reef track
HW14	26.0944	−80.1163	Coastal area, inlet

**Figure 1:**
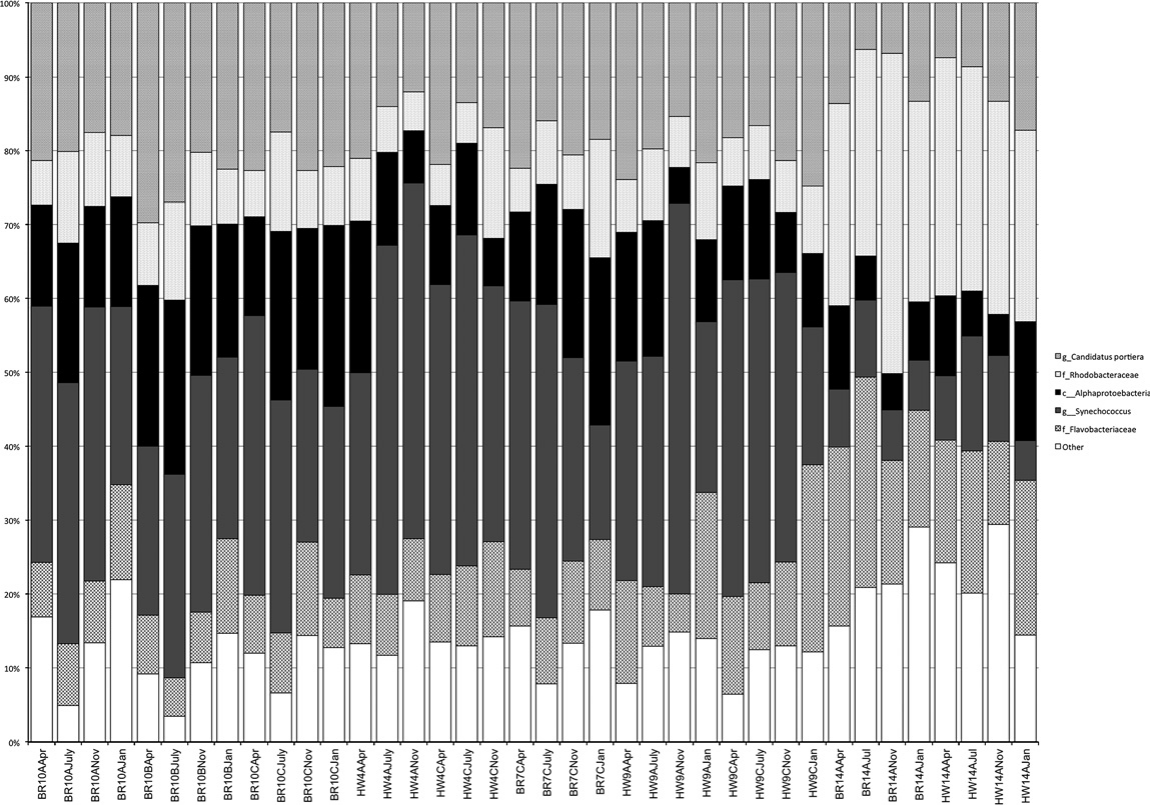
Taxonomic summary of the most abundant taxa (>1%) across all sites and seawater samples. Operational taxonomic units (OTUs) were determined through QIIME (Quantitative Insights into Microbial Ecology) analysis. The p__, c__, o__, f__, represent taxonomic rank.

The approximate latitude and longitude coordinates of seawater sample sites were obtained from NOAA FACE cruises. Site IDs correspond to individual sample IDs shown in subsequent Figures1 and 3.

### 16S rRNA amplicon sequence analysis

Barcoded universal primers, MIDf-515F and 806rc (Caporaso et al. 2011), were utilized for PCR amplification and sequencing each water sample's total metagenomic DNA. A BioRad MJ Mini thermal cycler (Bio-Rad, Berkeley, CA) was used to amplify the V4 hypervariable region, a region of high taxonomic identification confidence (Wang et al. 2007). PCR protocol followed (Caporaso et al. 2011) and generated PCR products between 300 and 350 base pairs. Amplicons were cleaned and sequenced on the 454 GS FLX Titanium platform through the University of Kentucky. The 16S ribosomal RNA sequences were deposited into the Sequence Read Archive under the accession number SRP046214.

The sequences were analyzed using Quantitative Insights into Microbial Ecology (QIIME v.1.8.0) (Caporaso et al. 2010). Quality checking encompassed denoising sequences with Denoiser (Reeder and Knight 2010), and removing chimeric operational taxonomic units (OTUs) with ChimeraSlayer (Haas et al. 2011). OTUs were assigned using uclust (Edgar 2010) with 97% similarity and open reference OTU picking. Taxonomic assignments were made using the UCLUST taxonomic assigner (Rideout et al. 2014) with a 90% confidence cutoff and the Greengenes 13_8 reference database (DeSantis et al. 2006; McDonald et al. 2012a). Sequences were aligned using the Greengenes reference alignment (DeSantis et al. 2006) and PyNastv1.2 (Caporaso et al. 2010). A phylogenetic tree was created with FastTree (Price et al. 2010) and a resulting OTU table. The OTU table was run through the alpha_diversity.py script to generate Shannon diversity and abundance-based coverage estimation (ACE). ACE characterizes alpha diversity across samples and uses the frequency of OTUs with 10 observations or less to estimate the diversity per sample (Chao and Lee 1992; Chao and Shen 2004). The values for Shannon and ACE diversity were averaged by site and plotted using Microsoft Excel.

Beta diversity analysis was conducted using weighted and unweighted Unifrac (Lozupone and Knight 2005; Lozupone et al. 2007; Hamady et al. 2010), a phylogenetically informed metric, to assess potential trends in shared OTU composition across sites (outfall, reef, inlet) and seasonality (wet, rainy). Principal coordinate analysis (PCoA) plots were generated using Emperor (Vazquez-Baeza et al. 2013), including color schemes based on associated metadata (site type, season) from a QIIME mapping file. We implemented ADONIS (compare_categories.py) to statistically test differences in the composition and relative abundance of different taxa of ecological communities (Anderson 2001).

To minimize bias associated with differences in sampling depth, OTU counts were rarefied to 1564 sequences per sample prior to downstream analysis and collated in QIIME (Caporaso et al. 2010). At each taxonomic level (phylum through genus), we determined taxa that were shared among marine environments (inlet, outfall, and reef), and unique to each marine environment. We also arranged taxonomic groups representing at least 0.1% (on average) of 16S rRNA sequences per sample. Furthermore, we performed hierarchical clustering of 16S rRNA profiles summarized at all available taxonomic levels using furthest neighbor clustering with a Euclidean distance metric. This was done using the Skiff tool in CloVR (Angiuoli et al. 2011).

### Statistical analysis

In QIIME 1.8.0, the compare_categories.py script was used to determine the analysis of variance using distance matrices (ADONIS) and 999 permutations. The parameters tested were depth, season, and location (reef, outfall, and inlet). SAS was used to perform a multiple least squares regression analysis, using available environmental metadata as the independent variables (physical oceanographic metadata collected by ship GPS and by the SBE 19V2 CTD sensor suite as described above) (Table S1). A least squares regression model was run separately on each of the most abundant taxa in the dataset (Table3). A backward selection process was used with model retention at *α* = 0.10.

## Results

### 16S rRNA community analysis

A total of 38 seawater samples were collected and analyzed for bacterioplankton community variation over four consecutive quarters starting from April 2011 until January 2012.

DNA pyrosequencing of these samples resulted in 393,545 raw sequences, which further reduced to 227,499 (Table S4) after denoising and chimera checking. Across all samples, the number of OTUs ranged from 321 to 19,998, with a mean of 5986 counts. Multiple rarefaction analysis supported the finding that at least 30 samples yielded >2500 sequences (Table S4). From these data, a total of 4447 OTUs were generated after comparison with the Greengenes database (OTU table from Greengenes generated March 2014).

### Alpha diversity

**Figure 2:**
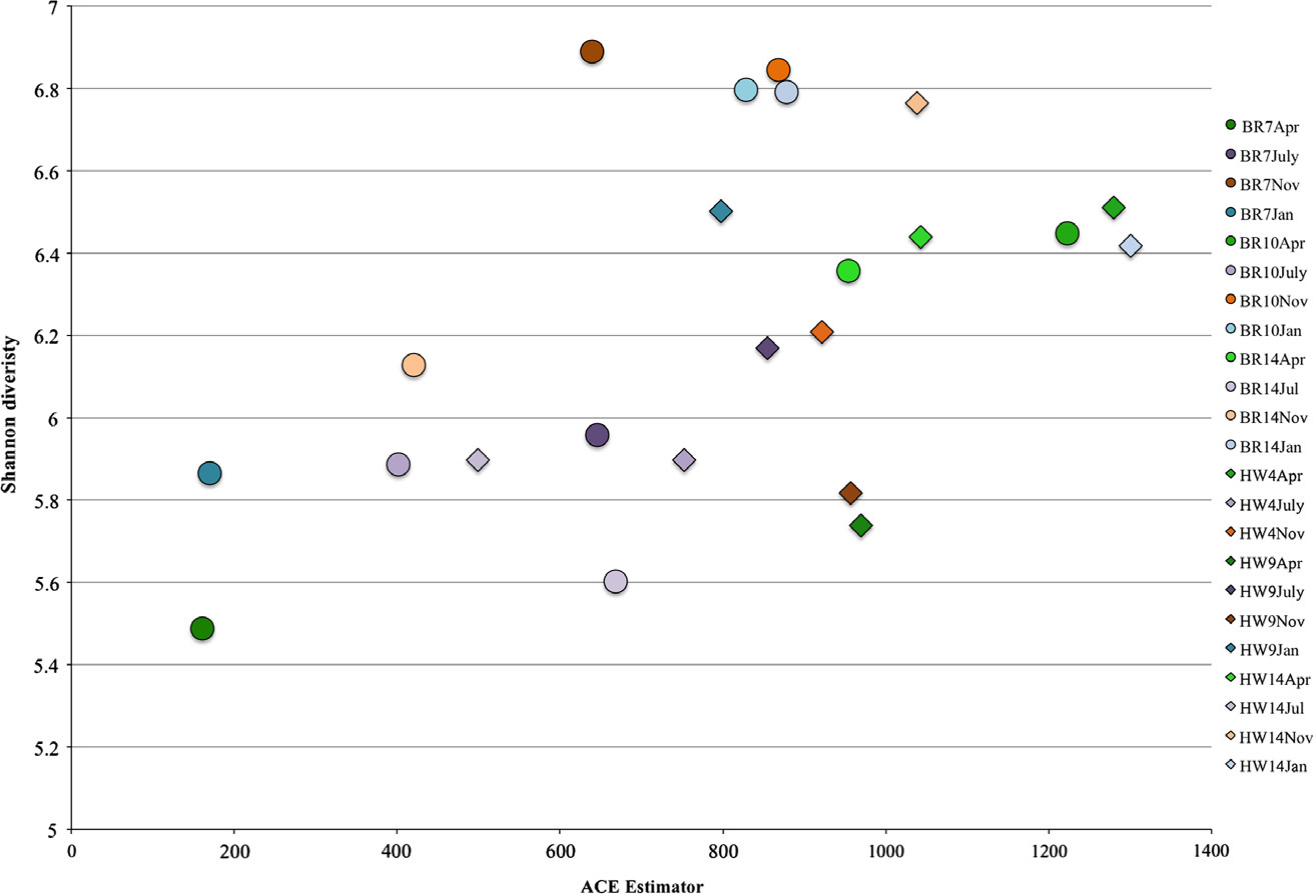
Shannon and abundance-based coverage estimation (ACE) diversity plot. Each seawater community appears similarly diverse. The average diversity for each site was taken. According to the abundance coverage estimator, certain samples (BR7Apr, BR7Jan, and BR10July) show lower richness despite being equally diverse. Fewer operational taxonomic units (OTUs) (<2000) were observed for these samples. BR7Nov, BR10Jan, BR10Nov, and BR14July showed the most Shannon diversity despite modest ACE values.

**Figure 3:**
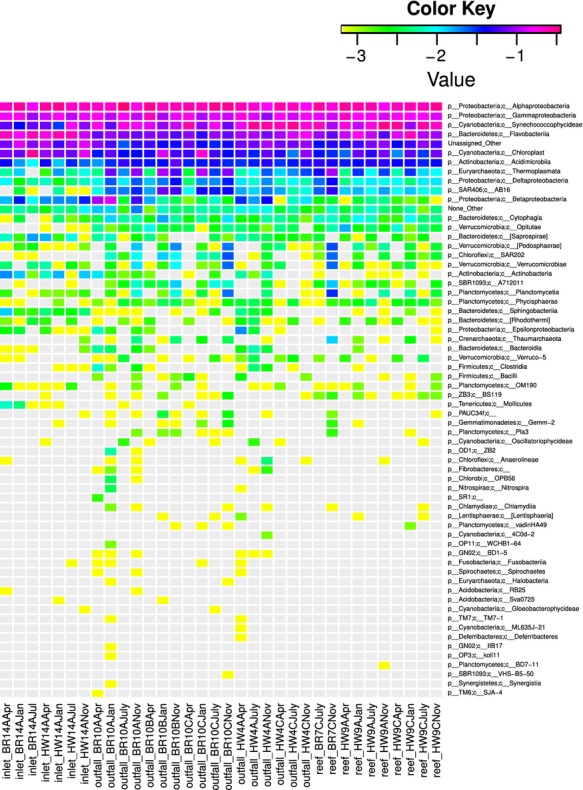
Hierarchical clustering of bacterioplankton 16S profiles at the class level. Heatmap values reflect log-normalized proportions (e.g., −1% to 10%, −2% to 1%, −3% to 0.1%) and were generated using the skiff tool in CloVR (Angiuoli et al. 2011). Relative microbial abundance is shown by the spectrum of colors on a logarithmic scale. For example, a more reddish color indicates higher abundance, while yellow indicates less common taxa.

Alpha diversity gauges bacterial variation within a specific site (Whittaker 1972; Sepkoski 1988), such as the abundant taxa associated with each site type. The most abundant community members were present in numbers greater than or equal to 1% across all samples (Fig.1). In addition, the Shannon and ACE plot indicated a relatively low bacterioplankton diversity range across all samples, with the greatest diversity found at reefs and outfalls (Fig.2). To further compare diversity across all samples and sites, abundance heatmaps from phylum to family levels were generated (Figs.3, S2, and S3). The heatmaps visualize an unsupervised clustering of samples and further emphasize the most common classes, which in this study were the Cyanobacteria, Flavobacteria, Alphaproteobacteria, Gammaproteobacteria, and several unidentified bacterial taxa.

### Abundant taxa

The most abundant bacterial taxa we found in this study included Cyanobacteria, Bacteroidetes, and Proteobacteria (Table2). Our results were consistent with earlier literature (Giovannoni and Rappé 2000; DeLong 2009) and parallel studies in our laboratory, which have sequenced multiple southeast Florida seawater control samples as part of marine disease (Negandhi et al. 2010; R. Mulheron, unpubl. data; C. Walton, unpubl. data) and pollution research (Cuvelier et al. 2015).

**Table 2:** The most abundant taxa (>1%) across all samples

	Flavobacteriaceae (%)	Synechococcus (%)	Alphaproteobacteria (%)	Rhodobacteraceae (%)	Candidatus Portiera (%)	Pelagibacteraceae (%)
HW4AApr	5.032	14.832	11.121	4.564	11.416	6.953
HW4AJuly	4.913	28.322	7.517	3.726	8.375	6.462
HW4ANov	4.292	24.565	3.625	2.697	6.119	9.194
HW4CApr	5.036	21.717	5.945	3.042	12.126	14.426
HW4CJuly	6.819	28.279	7.782	3.490	8.504	9.627
HW4CNov	7.583	20.317	3.804	8.798	9.934	3.170
BR10AApr	3.505	16.554	6.560	2.851	10.208	9.084
BR10AJuly	4.412	18.542	9.910	6.522	10.550	16.113
BR10ANov	3.310	14.718	5.430	3.953	6.954	7.192
BR10AJan	3.538	6.601	4.075	2.274	4.927	12.571
BR10BApr	4.583	13.122	12.487	4.904	17.077	8.135
BR10BJuly	2.804	14.953	12.773	7.165	14.642	9.346
BR10BNov	2.828	13.178	8.331	4.090	8.331	6.993
BR10BJan	4.332	8.326	6.089	2.503	7.634	10.811
BR10CApr	4.816	23.490	8.358	3.866	14.074	9.933
BR10CJuly	4.054	15.687	11.376	6.714	8.701	10.816
BR10CNov	4.037	7.499	6.119	2.507	7.266	19.397
BR10CJan	2.282	8.949	8.438	2.733	7.628	8.228
HW9AApr	9.094	19.414	11.354	4.636	15.612	9.985
HW9AJuly	4.820	18.788	11.025	5.830	11.861	5.368
HW9ANov	3.074	31.865	2.908	4.155	9.265	6.107
HW9AJan	9.680	11.264	5.468	5.079	10.577	12.997
HW9CApr	8.758	28.528	8.407	4.382	12.118	10.341
HW9CJuly	5.705	25.945	8.503	4.651	10.465	8.430
HW9CNov	5.582	19.363	4.017	3.441	10.557	22.158
HW9CJan	12.507	9.236	4.945	4.500	12.271	11.774
BR7CApr	4.607	21.951	7.317	3.523	13.550	7.317
BR7CJuly	4.817	22.757	8.727	4.618	8.573	10.694
BR7CNov	3.774	9.376	6.834	2.503	6.992	8.661
BR7CJan	2.477	4.025	5.882	4.180	4.799	10.526
HW14AApr	7.505	3.930	4.873	14.549	3.377	5.278
HW14AJul	9.413	7.616	2.980	14.853	4.257	3.737
HW14ANov	5.184	5.412	2.558	13.359	6.166	4.659
HW14AJan	8.495	2.166	6.529	10.526	6.999	13.433
BR14AApr	10.751	3.507	5.004	12.206	6.050	5.642
BR14AJul	10.976	4.017	2.296	10.760	2.439	1.327
BR14ANov	7.054	2.905	2.075	18.257	2.905	0.553
BR14AJan	7.158	3.085	3.548	12.311	6.048	11.139

In the class Cyanobacteria, the most abundant OTU was the genus *Synechococcus*, a globally distributed phytoplankton (Palenik et al. 2003). Unique features of *Synechococcus* include its ability to utilize organic nitrogen and phosphorus and adapt to oligotrophic environments, though less abundant than a related genus *Prochlorococcus* (Moore et al. 1995; Palenik et al. 2003).

Another abundant microbe found in southeast Florida waters was Pelagibacteraceae, formerly known as SAR11. This was one of the first bacterial clades identified using cultivation-independent techniques and found to be well adapted to low nutrient environments (Morris et al. 2002; Rappé et al. 2002). Pelagibacteraceae have been found in every aquatic 16S clone library since their discovery, thought to account for over 18% of the abundance in photic waters (Morris et al. 2002). Our data show high numbers of Pelagibacteraceae across all water samples, and a correlation with chlorophyll-a (Table3).

**Table 3:** Multiple regression analysis output of metadata to most abundant taxa using SAS software

	Flavobacteriaceae	Synechococcus	Rhodobacteraceae	Candidatus Portiera	Pelagibacteracaeae
*R*^2^	0.4817	0.5882	0.9347	0.7431	0.2745
Date	NS	0.0015	0.0117	<0.0001	NS
Depth	NS	NS	0.0616	NS	NS
Time	0.0099	NS	NS	0.0400	NS
Salinity	0.0088	NS	<0.0001	0.0069	NS
Temperature	NS	NS	<0.0001	0.0099	NS
pH	NS	NS	0.0414	NS	NS
O_2_ saturation	NS	NS	<0.0001	NS	NS
ORP	NS	NS	NS	NS	NS
Conductivity	NS	0.0092	NS	NS	NS
Chlorophyll-a	NS	NS	NS	0.0632	0.0007
Phaeopigment	<0.0001	0.0155	NS	0.0014	NS
TSS	NS	0.0427	<0.0001	NS	NS
Total nitrogen	NS	0.0804	NS	0.004	NS
Nitrate	NS	0.0830	0.0015	NS	NS
Ammonium	NS	NS	NS	NS	NS
Phosphorus	NS	NS	NS	NS	NS
Silica	NS	0.0035	NS	NS	NS

*R*^2^ value shows how much of the variation of dependent variable is explained by the independent variables in the final regression equation and thus how well the model fits the data. A higher *R*^2^ value means that the model fits the data well. A comparison to the percentage of the abundant taxa was made to environmental metadata was performed. The *P*-value threshold was set at 0.1. Any parameter above 0.1 was discarded. Values greater than 0.1 on this table are considered to be approaching statistical significance and are included. No parameter was consistent among all abundant bacteria. The *P*-values were obtained from SAS. NS, not significant, *P* > 0.1. ORP, oxygen reduction potential; TSS, total suspended solids.

Another abundant OTU in our dataset matched to the genus *Candidatus* Portiera, an endosymbiont of the whitefly (Bing et al. 2013). *Candidatus* Portiera showed a significant relationship with time, salinity, temperature, phaeopigment, and total nitrogen (Table3). The presence of this taxon is likely an error in the Greengenes database, and will likely be resolved in the next release of Greengenes (McDonald and Hugenholtz 2014). This taxon belongs within the order Oceanospirillales, based on previous draft genome data (Jiang et al. 2012), and are common marine Gammaproteobacteria (Giovannoni and Rappé 2000) with the ability to degrade hydrocarbons (Lamendella et al. 2014).

Flavobacteriaceae commonly occurs in marine samples and break down large molecules like chitin, DNA (Giovannoni and Rappé 2000), and algal degradation products (Gómez-Pereira et al. 2010). Flavobacteriaceae are often associated with higher primary production, namely in colder waters, and often dominate marine picoplankton communities (Gómez-Pereira et al. 2010). The family *Flavobacteriaceae* (*R*^2^ = 0.4817) showed a statistically significant relationship with time of day, salinity, and phaeopigments, an algal degradation product (Abdell and Bowman 2005). Instead, like other Flavobacteriaceae, they can hydrolyze complex molecules like casein and gelatin (Lee et al. 2010).

Family Rhodobacteraceae (Class Alphaproteobacteria) also appeared among the abundant taxa across most samples. They are rapid surface colonizers (Dang et al. 2008) and are purple, photosynthetic bacteria. A significant environmental link with Rhodobacteraceae was temperature (Table3), previously seen in a study by Stratil et al. (2013). Rhodobacteraceae showed a significant relationship with salinity, oxygen saturation, pH, TSS, and nitrate concentration according to multiple least squares analysis (Table3, and see below).

### Site-dependent abundant taxa

An abundant bacterial taxon identified on the reefs and outfalls was the family OCS155, which belongs to the order Acidimicrobiales and the family Pelagibacteraceae (Figs. S4 and S5). SAR406, a common clade in open ocean waters (Biers et al. 2009), Deltaproteobacteria, and archaea Thermoplasmata, which can be found in a variety of habitats (DeLong and Pace 2001; Pires et al. 2012) including surface waters (DeLong 2006), represented other taxa that stood out at reef and outfall sites (Fig.3).

Planctomycetes occurred in elevated numbers at the inlets in this study (0.13–4.05%), which is consistent with previous isolations from various aquatic ecosystems such as a prawn gut (Fuerst et al. 1997) and at cold-water *Lophelia* coral reefs (Neulinger et al. 2008; Kellog et al. 2009). They show correlations with algal blooms, due to their ability to subside on algal degradation products (Pizzetti et al. 2011). These bacteria were found in elevated numbers at the Broward reef sites (Figs.3 and S3), but interestingly, with no concomitant increase in chlorophyll-a and phaeopigments.

The inlet specific taxa also included abundant family Cryomorphaceae, order Stramenopiles, family OM60 (order Alteromonadales), and Lentisphirae. Cryomorphaceae (Alonso et al. 2007) are members of the Flavobacteriales order, and can hydrolyze complex molecules like casein and gelatin for energy (Lee et al. 2010). Stramenopiles are photosynthetic eukaryotes and were found across all samples, with highest abundance occurring at the inlets. Stramenopiles occur within the cyanobacterial radiation (Giovannoni et al. 1988) and show close similarity to prokaryotic 16S rRNA of the unicellular, nitrogen-fixing cyanobacteria (Zablen et al. 1975; Falcón et al. 2010). While previous studies have found Lentisphaerae in landfill leachate (Chouari et al. 2005; Limam et al. 2010) and marine datasets (Cho et al. 2004), Lentisphaerae were found in the inlet samples. A higher presence of Betaproteobacteria was also found at the inlets (Figs.3, S3, and S4). Previous clone and culturing studies have shown the presence of Betaproteobacteria in both the open ocean (Rusch et al. 2007), and in freshwater ecosystems while a recent study by Kelly et al. (2014) found more Betaproteobacteria on algal dominated reefs.

At the outfall sites, the phylum Bacteroidetes was in high abundance (Figs.3, S2, and S3). Bacteroidetes comprise a large proportion of human gut flora (Qin et al. 2010) and are common in aquatic ecosystems (Kirchman 2002; Lydell et al. 2004; Abdell and Bowman 2005). They belong to the Cytophaga–Flavobacteria–Bacteroidetes (CFB) cluster, which is typically associated with particulate matter, though there are many free-living species (Abdell and Bowman 2005). Chloroflexi, Nitrospirae, and Firmicutes sequences were also identified in outfall communities, along with SBR1093, a sludge-associated Chlorobi-like taxon (Rappé and Giovannoni 2003) (Figs. S2 and S5). We also found Thiotrichales, filamentous, sulfur-oxidizing microbes, which includes the genus *Beggiatoa*, a mat-forming microbe which may indicate organic pollution (Chet and Mitchell 1975; Fenchel and Bernard 1995; Elliot et al. 2006).

### Beta diversity

Beta diversity measures the diversity among groups (Whittaker 1972; Sepkoski 1988). In combination with PCoA, this approach compared groups, such as site type and season, based on actual 16S rRNA sequence counts or phylogenetic analysis.

**Figure 4:**
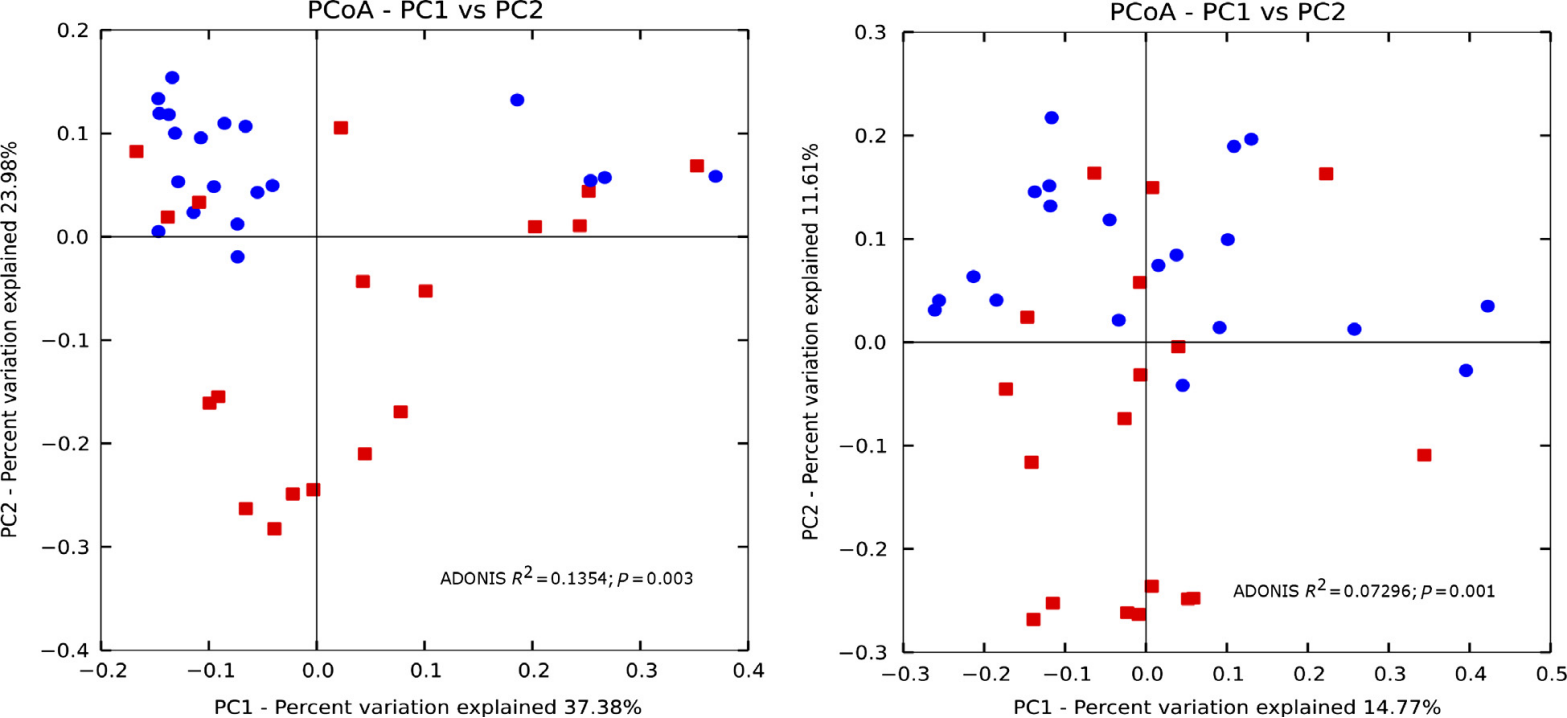
Principal coordinate analysis (PCoA) plot of the samples by season (rainy vs. dry) using the weighted (left) and unweighted (right) Unifrac measurement. Dry, red; rainy, blue.

Bacterioplankton composition showed significant changes between seasons. In southeast Florida, the fall and winter months, October to early March, are considered dry season. The spring and summer months, April–September, typically have more rainfall. Total rainfall during our four quarter sampling period (April 2011–January 2012) generally conformed to the dry and wet season definitions. Rainfall in the Fort Lauderdale area had the following monthly patterns (shown in millimeters): April 2011 (84.07), July (197.6), November 2011 (73.6), and January 2012 (13.97) (http://www.usclimatedata.com/climate/florida/united-states/3179). However, rainfall patterns just 3 days before each collection date showed slight deviations within the monthly records. For example, the high November rainfall reading of 73.6 mm, diminished to only 2.3 mm before collection (Table S5). Bacterioplankton members showed significant clustering by weighted UniFrac analysis (Fig.4) with at least one rainy (blue) outlier explicable by lack of rainfall before the collection date (13 April 13 2011). Some bacterial and archaeal classes showed significant seasonal changes between wet and dry seasons, regardless of site (ADONIS *R*^2^ = 0.1354; *P* = 0.003) allowing us to reject the null hypothesis of there being no change due to seasonal (rainfall) variability. We measured differences with the unweighted Unifrac distance matrix, which also showed significant changes (ADONIS, *R*^2^ = 0.07296, *P* = 0.001).

**Figure 5:**
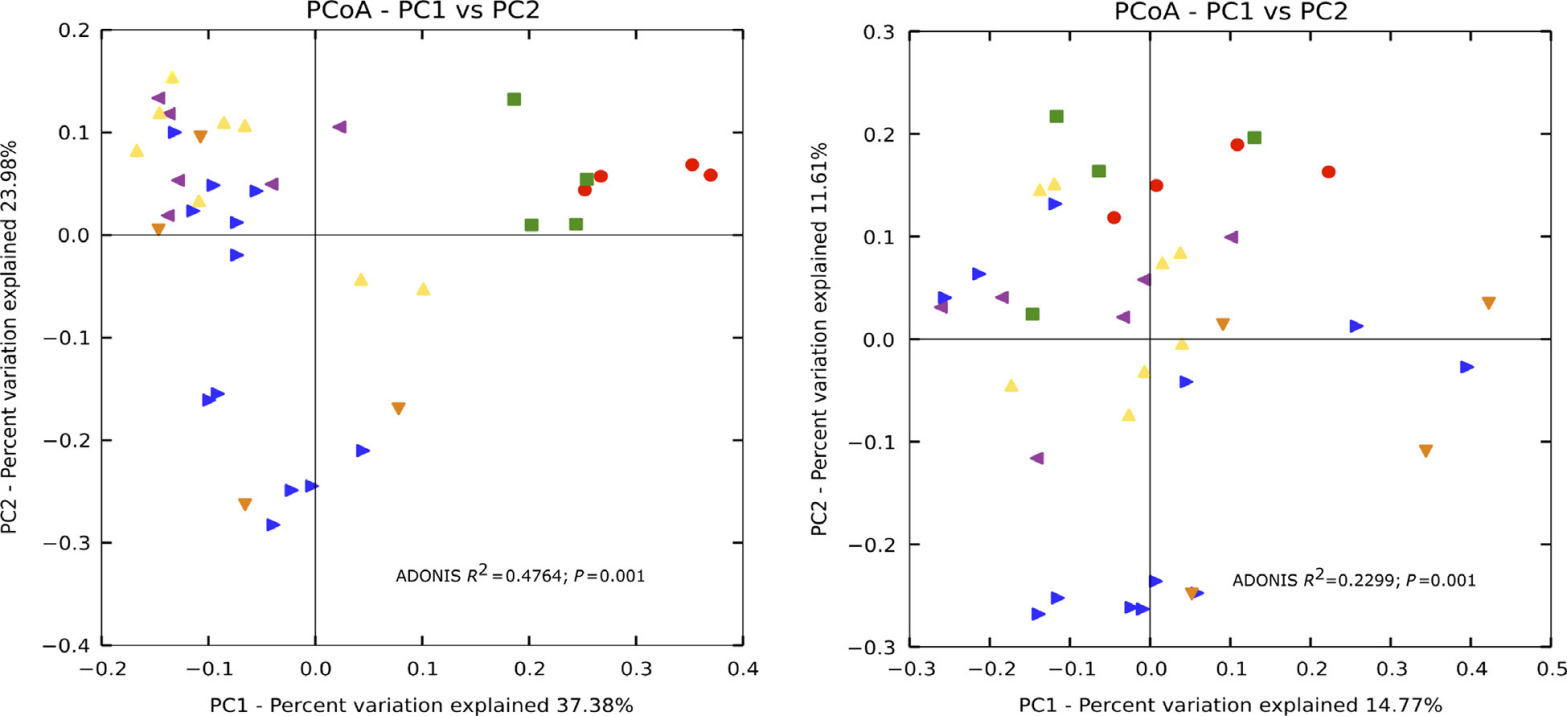
Principal coordinate analysis (PCoA) plot of the samples by site type (outfall, inlet, reef) using the weighted (left) and unweighted (right) Unifrac measurement. Red, BRinlet; green, HWinlet; yellow, HWreef; blue, BRoutfall; orange, BRreef; purple, HWoutfall.

**Figure 6:**
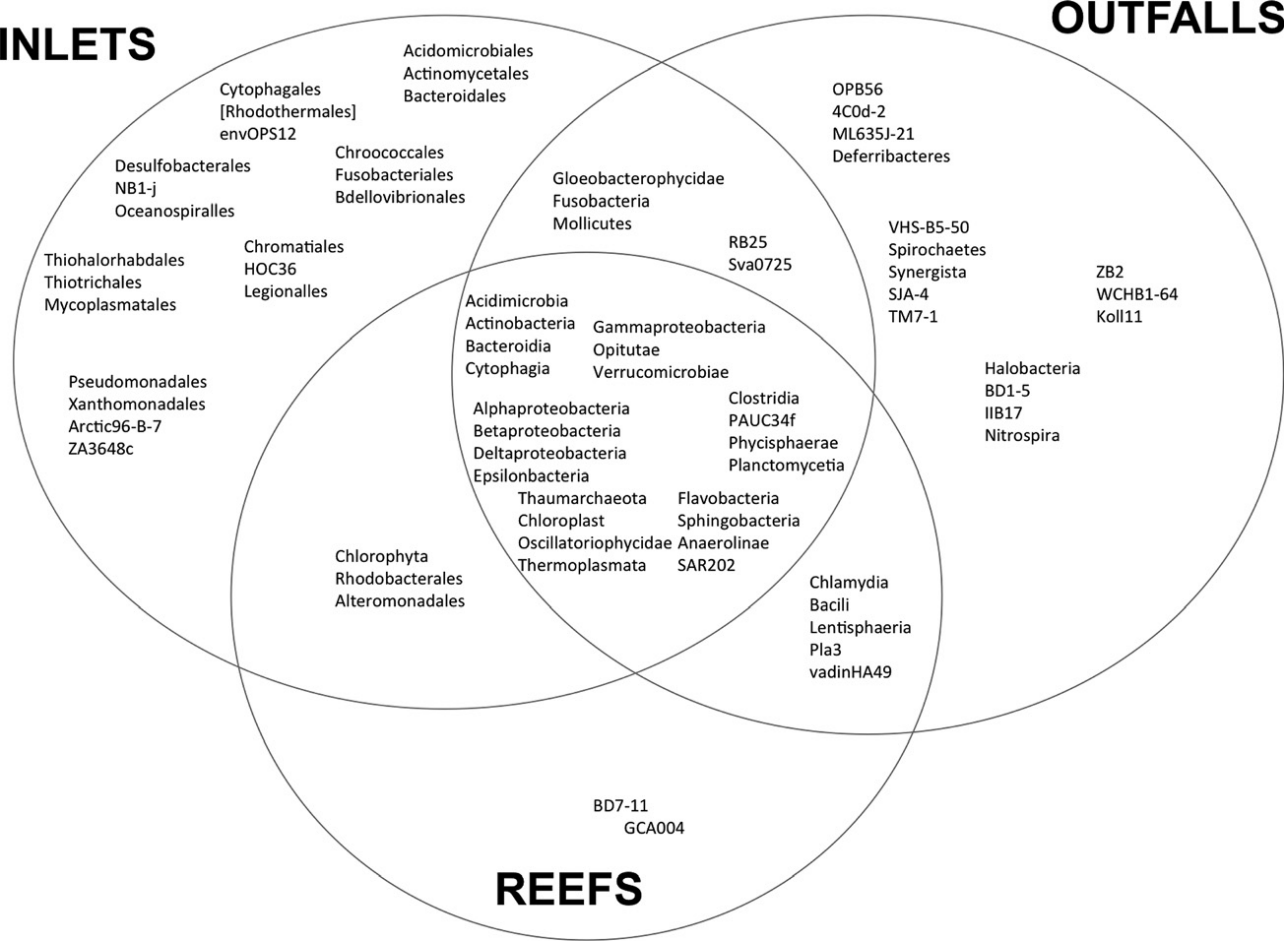
Venn diagram of the shared and unique taxa at class level across each site type. Displayed taxa represented at least 0.1% of sequences per sample.

**Figure 7:**
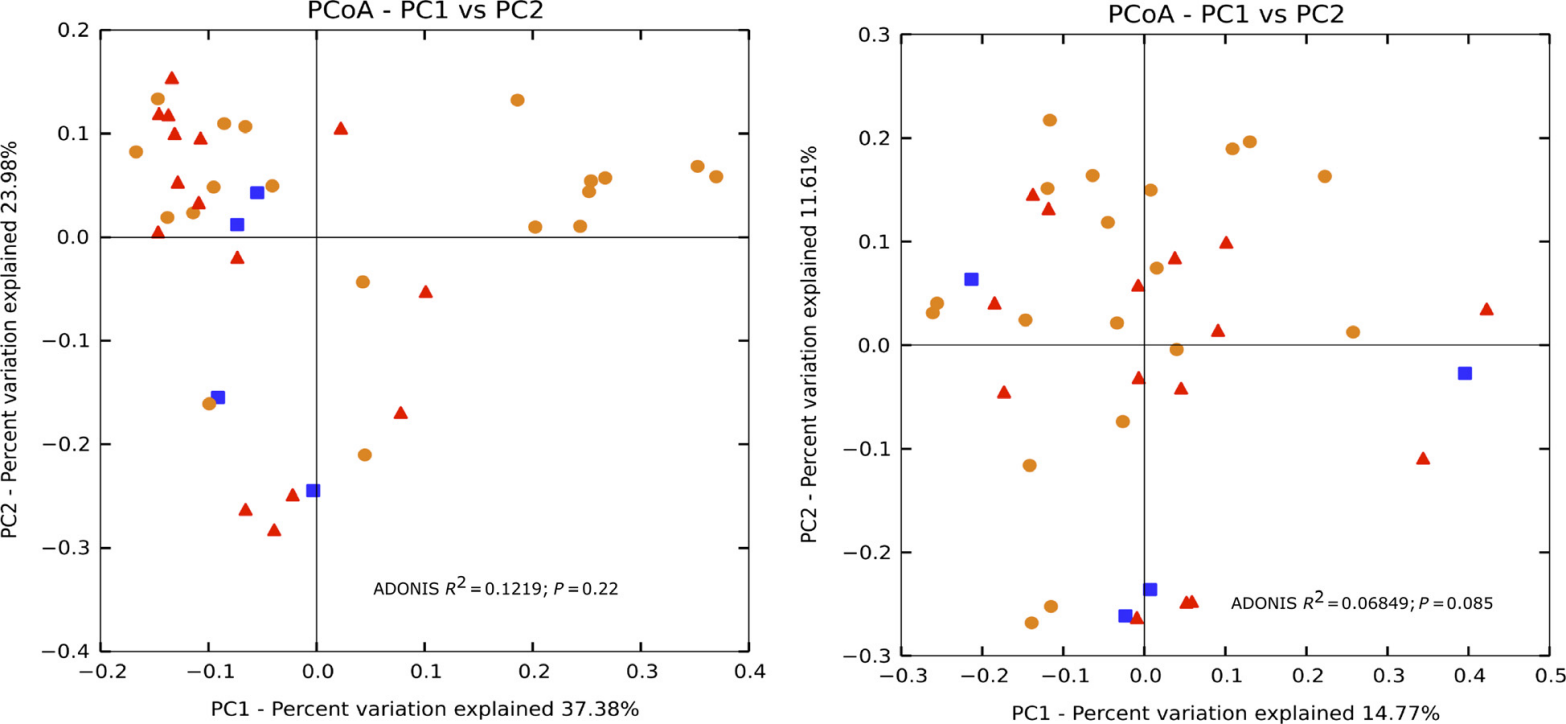
Principal coordinate analysis (PCoA) plot of the samples by depth (surface, mid-depth, bottom) using the weighted (left) and unweighted (right) Unifrac measurement. Orange, surface; blue, mid-depth; red, bottom.

We also examined the spatial changes in beta diversity by site type: reefs, outfalls, and inlets. The beta diversity analysis was not normalized (McMurdie and Holmes 2014). The results of the PCoA using the weighted Unifrac distance metric showed a distinct clustering of the Broward (red) and Hollywood (green) inlets (Fig.5). Statistical significant differences were found comparing the site type by ADONIS (*R*^2^ = 0.4764; *P* = 0.001), rejecting the null hypothesis of no changes occurring with site type. The unweighted Unifrac matrix was compared (ADONIS, *R*^2^ = 0.2299, *P* = 0.001). The bacterial communities of the Hollywood and Broward reefs and outfalls cluster according to site type. A comparison of the similar communities found at each site can be seen in Figure6. Significant changes found with depth using the weighted Unifrac matrix (ADONIS, *R*^2^ = 0.1219; *P* = 0.022), but not the unweighted matrix (ADONIS, *R*^2^ = 0.06849, *P* = 0.085) (Fig.7). Depth was calculated using nonnumerical values (surface, mid-depth, and bottom).

A multiple least squares regression analysis was performed separately on the four most abundant bacterial taxa and various seawater chemical parameters (Table3). Although multiple seawater parameters were measured (see Experimental Procedures section), only the following showed significant associations with microbial abundance. Genus *Synechococcus* (*R*^2^ = 0.5882) showed a significant relationship with conductivity (*P* < 0.05) and phaeopigments (*P* < 0.05), TSS (*P* < 0.05), and silica (*P* < 0.05). Family *Flavobacteriaceae* (*R*^2^ = 0.4817) showed a statistically significant relationship with time (*P* < 0.05), salinity (*P* < 0.05), and phaeopigments (*P* < 0.0001). *Rhodobacteraceae* (*R*^2^ = 0.9347) had the highest number of significant variables, showing significant relationships with sampling date (*P* < 0.05), salinity (*P* < 0.0001), temperature (*P* < 0.0001), oxygen saturation (*P* < 0.0001), pH (*P* = 0.0414), TSS (*P* < 0.0001), and nitrate (*P *= 0.0015). The genus *Candidatus* Portiera (*R*^2^ = 0.7431) showed a significant relationship with sampling date (*P* < 0.0001), time (*P* < 0.05), salinity (*P* < 0.05), temperature (*P* < 0.05), phaeopigments (*P* < 0.05), and total nitrogen (*P* < 0.05).

## Discussion

The health and dynamics of most ecosystems depend on microbial life. Continued exploration of the microbiomes will greatly benefit ecosystem-monitoring projects by delineating specific microbial taxa, their interactions, and their response to natural and anthropogenic impacts (DeLong 2009). DNA sequence data, such as that obtained via high-throughput sequencing, can detect rare species, (Pedrós-Alió, 2012) which may serve as indicators of environmental change (Lynch and Lande 1993; Sogin et al. 2006; Gilbert et al. 2010a,b; Kirchman et al. 2010). Our data suggest that bacterioplankton assemblages can be coupled with seasonal (rainfall) patterns, which could affect land-based runoff (Brownwell et al. 2007; Jackson et al. 2014). The link between rainfall patterns, land runoff, and its affect on aquatic microbiomes warrants further investigation.

Previous coastal water quality monitoring studies have focused on target organisms, usually coliforms and FIB (Finkl and Charlier 2003; Shibata et al. 2004; Brownwell et al. 2007; Hartz et al. 2008; Harwood et al. 2009; Wright et al. 2009; Abdelzaher et al. 2010; Staley et al. 2012), such as the work done by the FACE program, utilizing both culture-based and molecular quantitative PCR techniques (Carsey et al., unpubl. data).

The current community genomic study reported here has produced a broad taxonomic survey of marine bacterial communities in southeast Florida waters. Dominant classes of bacteria detected included *Cyanobacteria*,*Alphaproteobacteria*,*Gammaproteobacteria*, and *Flavobacteria*, which were abundant across all samples. A causal relationship for the occurrence of archaeal groups could not be discerned at this time.

With regard to depth, previous work by Caro-Quintero and Konstantinidis (2012) found changes in the microbial structure below the photic zone. Since the samples in our study were found well within the photic zone, major microbial composition shifts with depth were not expected and did not occur. Likely, the results showed by the ADONIS analysis of the weighted and unweighted Unifrac suggest that there may be a significant difference in the presence/absence of certain OTUs. The mid-depth was only analyzed for the Broward samples, skewing the results, and may have been a contributing factor for this discrepancy.

Microbial composition changed significantly across seasons in this study. The clustering by seasons may be due to southeast Florida having distinct wet (June–September) and dry seasons (Banks et al. 2008). Depending on the time of year, the salinity of inlet plumes will also be lower in the wet season (June–September). Although we did not perform functional or diel characterizations, our taxonomic identifications are consistent with recent functional studies of coastal bacterioplankton (Gifford et al. 2014).

Although pathogenic bacterial sequences rarely appeared in the current dataset, the following genera were detected: *Vibrio* (1%), *Staphylococcus* (>0.1%), *Campylobacter* (0.5%), and *Clostridium* (>0.01%). Also, we noted an increased occurrence of Verrucomicrobiales at the Broward outfalls and reefs while Actinobacteria and Tenericutes appeared higher at the inlets (Figs.3, S2–S6). These findings alleviate human health concerns, but may still have significance in the context of growing marine disease concerns (Burge et al. 2014). For example, concurrent studies of marine symbiont microbiomes in our laboratory have associated some of the above taxa, such as Tenericutes, *Verrucomicrobia*, and Planctomycetes with intermittent “sponge orange band” (SOB) disease that can afflict the iconic giant barrel sponge *Xestospongia muta* found throughout the Florida reef tract (Mulheron et al., unpubl. data; Angermeier et al. 2011).

The weighted PCoA analysis showed microbial communities significantly grouped by site. The Venn diagram (Fig.6) further summarizes and delineates the microbial community differences between sites, mostly among rare classes. Both Hollywood and Broward (Pompano Beach) inlet microbial communities were distinct from the outfalls and reef communities and surprisingly grouped together despite being separated by ∽20 km. Inlets directly connect the ICW with the Atlantic Ocean, and are highly subject to tides. Conversely, their outgoing tidal plumes can influence nutrient loads, sedimentation, and coastal circulation in surrounding waters. The height of the tides can also affect overall inlet contribution to reefs (Banks et al. 2008). Coastal canals indent residential neighborhoods and can eventually funnel runoff or anthropogenic pollutants to the ICW and the inlets (Futch et al. 2011).

The regression analyses indicated that some of the most abundant microbial taxa appear significantly correlated with specific components in the seawater (Table S1). For example, it is expected that *Synechococcus* would show a link to bloom-related phaeopigments, while the high number of variables associated with *Rhodobacteraceae* abundance is consistent with this family encompassing the physiologically diverse, marine Roseobacter clade (Buchan et al. 2005). Because these marine environmental features varied by site, they provide starting points for future hypothesis-testing and tracking of variables that may most strongly influence bacterioplankton community structure.

Port Everglades inlet in Fort Lauderdale, FL, is 245-m wide and 15-m deep, and has its own inherent currents (Stamates et al. 2013; Carsey et al. in press[Bibr b19]). Pompano Beach's Hillsboro Inlet is 94-m wide and 3-m deep. The relatively shallow inlet waters may more easily translate small changes in temperature, salinity, nutrients, etc., to their microbial communities. Moreover, inlet microbiome content may be affected by heavy runoff (Brownwell et al. 2007), boat traffic, industry, and proximity to recreational beaches.

Perhaps, unexpectedly, outfall sites showed little distinction between their microbiomes and the reefs’. Secondarily treated wastewater is discharged by a series of outfalls, two of which are located within Broward County, the Broward Outfall and Hollywood Outfall (Futch et al. 2011). Secondary treatment refers to wastewater that was strained of large solids, then disinfected and dechlorinated before being discharged into the environment (Tchobanglous et al. 2003). The Broward outfall is located 2.2 km offshore at 32.6 m of depth (Koopman et al. 2006) and discharges 36 million gallons per day (MGD) of treated sewage (Carsey et al. 2010). The Hollywood outfall is located 3.1 km offshore, and situated at 28.3 m depth (Koopman et al. 2006), and discharges 40 MGD of treated sewage water (Carsey et al. 2010). The outfall plume spews mostly buoyant fresh water at the pipe (Carsey et al. in press[Bibr b19]), which mixes quickly with the surrounding seawater. Plume dilution takes about 2 min to move from the outfall to the surface, with strong mixing occurring as the water moves upward from the outfall. Further mixing occurs with the passing of the Florida Current. Effluent eventually rises to the surface about 10 m away from the outfall pipe (Koopman et al. 2006). With treatment, about 94% of TSS are removed meaning only 136 mg/L are expelled in the Hollywood outfall effluent. At the Broward outfall, 97% of TSS are removed, meaning 217 mg/L are expelled. Interestingly, besides Bacteroidetes, no FIB (e.g., *Enterococcus*) were detected at outfall sites. The only known spikes in nutrients occur at the surface of the outfall boil, but overall, these spikes did not significantly affect the surrounding waters (Carsey et al. in press[Bibr b19]). An explanation for the similar community profiles in outfall and reef microbiomes is that collections of samples near the top of the boil have already been diluted, or that the outfall boil was missed entirely.

The regulation of Florida wastewater outfalls is under the FDEP (http://www.dep.state.fl.us/water/wastewater) as part of the Clean Water Act of 1972 allowing the EPA to set effluent limits on an industry-wide basis and a water quality basis for the receiving waters (FDEP). Any discharge must obtain a permit from the National Pollutant Discharge Elimination System (NPDES), also developed under the Clean Water Act (1972). The long-term southeast Florida coastal policy aims to eliminate all wastewater discharges to the oceans by 2025 and to have 60% of those flows to be reused. Although an increase in stony corals was recently reported in southeast Florida (between 2011 and 2012) (EPA Office of Water 2013), these changes were determined to be nonsignificant (Gilliam et al. 2013). Also, coastal water quality standards, based on nitrogen and phosphorus, were not met (EPA Office of Water 2013). These increases in nutrient levels can correlate with algal blooms, and stress fragile ecosystems (Lapointe 1987; Lapointe and Bedford 2010). In 2013, the number of treatment plants receiving advanced water treatment or best available technology, recorded by equivalent dwelling units increased by 5%. This significantly exceeded the EPA strategic target and overall goal to provide adequate sewage treatment throughout the Florida Keys by December 2015. This study's annual profile of bacterioplankton communities associated with outfalls in their routine capacity also establishes a baseline for future comparisons.

The reefs and outfalls showed similar bacterial communities, and were different from the inlets. A possible reason for this could be meandering and reversing of the western portion of the Florida Current, resulting in counterclockwise rotating fronts and possible upwelling of deep, nutrient-rich water onto the shelf. Eddies may also form, which can range from 5 to 30 km in size and last 1–2 days (Carsey et al. in press.), with resulting currents mixing treated wastewater of the outfalls onto reefs. Land runoff and wastewater tend to be highly diluted downstream of the outfalls (Koopman et al. 2006).

## Conclusions

Overall, this project provides a baseline survey of bacterioplankton across two full seasons that can be useful for coastal management and environmental studies for a highly populated area. A longer and more frequent sampling regime is needed to fully assess microbial community structure dynamics for this region and should also include samples collected pre- and/or posttreatment from local wastewater plants. Long-term monitoring studies reflect the health and dynamics of an ecosystem (Gilliam 2010; http://ourfloridareefs.org). In the recent coral reef status report of Jackson et al. (2014), human activities such as land-based runoff or sewage releases continue to be emphasized as important factors in shaping community structure.

This high-throughput sequencing study represents one of the few to characterize some of the most common bacterioplankton of southeast Florida's wastewater outfalls, reefs, and inlets and complements current and future studies of benthic organism, meiofauna, and microorganisms of the outfalls, reefs, and inlets (Negandhi et al. 2010; Walker et al. 2012; White et al. 2012; Jackson et al. 2014). Marine organisms found in these environments are vital to reef health, and are directly affected by changes in water quality and bacterioplankton structure, particularly filter and suspension feeders such as sponges, corals, and oysters. These organisms can concentrate bacteria to levels and magnitudes higher than surrounding seawater (Massana and Pedrós-Alió 2008). Although the total marine area studied here may be relatively small (<200 km^2^), diverse environments and microhabitats in this area appear sufficient to structure microbial community diversity. In this study, pyrosequencing proved to give an adequately deep profile of bacterial diversity across the different local habitats, even though this sequencing technology has been recently eclipsed by higher throughput sequencing platforms (e.g., Illumina), which can generate millions more high-quality DNA reads at lower costs (Logares et al. 2012). However, this project helps establish next-generation DNA sequencing technologies as a vital tool for marine environmental monitoring in addition to the microbial culturing and quantitative PCR techniques utilized by the NOAA FACE program and similar entities.

## Supporting Information

Figure S1 Map of NOAA FACE collection sites. The sites used for this study are circled in red.

Figure S2 Hierarchical clustering of 16S profiles at the phylum level. Heatmap values reflect log-normalized proportions (e.g., −1% to 10%, −2% to 1%, −3% to 0.1%). As in Figure 3, relative microbial abundance is shown by the spectrum of colors on a logarithmic scale.

Figure S3 Hierarchical clustering of 16S profiles at the family level. Heatmap values reflect log-normalized proportions (e.g., −1% to 10%, −2% to 1%, −3% to 0.1%). As in Figure 3, relative microbial abundance is shown by the spectrum of colors on a logarithmic scale.

Figure S4 Taxonomic summary of the most abundant taxa (>1%) at the reef sites OTUs were determined through QIIME analysis.

Figure S5 Taxonomic summary of the most abundant taxa (>1%) at the outfall sites. OTUs were determined through QIIME analysis.

Figure S6 Taxonomic summary of the most abundant taxa (>1%) at the inlet sites. OTUs were determined through QIIME analysis.

Table S1 NOAA metadata table.

Table S2 Tides and collection times near Fort Lauderdale area outfalls, reefs, and Port Everglades inlet (see Fig. S1). For each date, tides were determined via http://tidesandcurrents.noaa.gov/.

Table S3 Tides and collection times near the Pompano Beach (BR-Broward) area outfalls, reefs, and Hillsboro inlet. For each date, tides were determined via http://tidesandcurrents.noaa.gov/.

Table S4 Operational taxonomic unit (OTU) summary table. Data were obtained post denoising and post chimera checking. The number of observations is the total number of unique OTUs across all samples. The counts refer to the number of total OTUs found in each sample, and the table density refers to a fraction of non-zero values (McDonald et al. 2012b).

Table S5 Precipitation record of Fort Lauderdale area during the study. Rainfall records in millimeters for the months and 3 days prior to each FACE sample collection date are summarized and based on the US Climate data (http://www.usclimatedata.com/climate/fort-lauderdale/florida/united-states/usfl0149).
