# Supplementary figures and images for: Dynamics of marine bacterial community diversity of the coastal waters of the reefs, inlets, and wastewater outfalls of southeast Florida
Source: Microbiologyopen. 2015 Mar 5;4(3):390–408. doi: 10.1002/mbo3.245 (PMC4475383; doi:10.1002/mbo3.245)

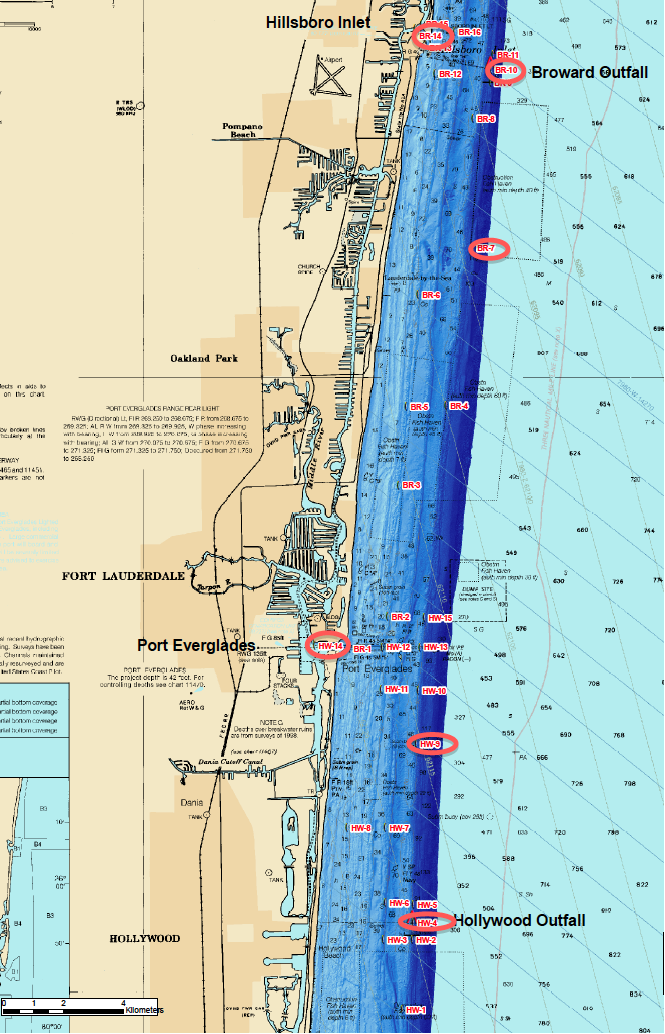

Supplement: Figure S1 — Map of NOAA FACE collection sites. The sites used for this study are circled in red. [file mbo30004-0390-sd1.tif]

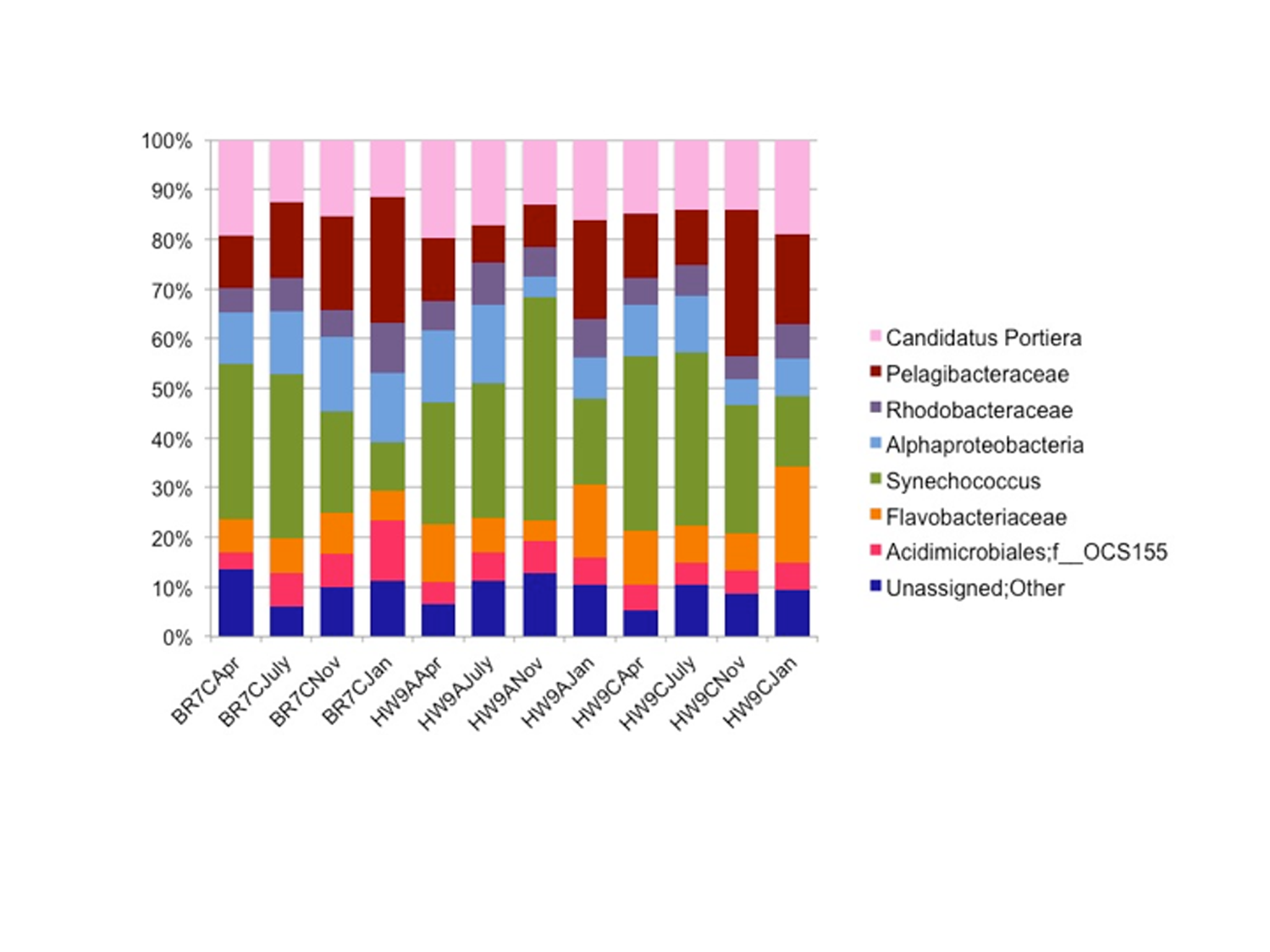

Supplement: Figure S4 — Taxonomic summary of the most abundant taxa (>1%) at the reef sites OTUs were determined through QIIME analysis. [file mbo30004-0390-sd4.tif]

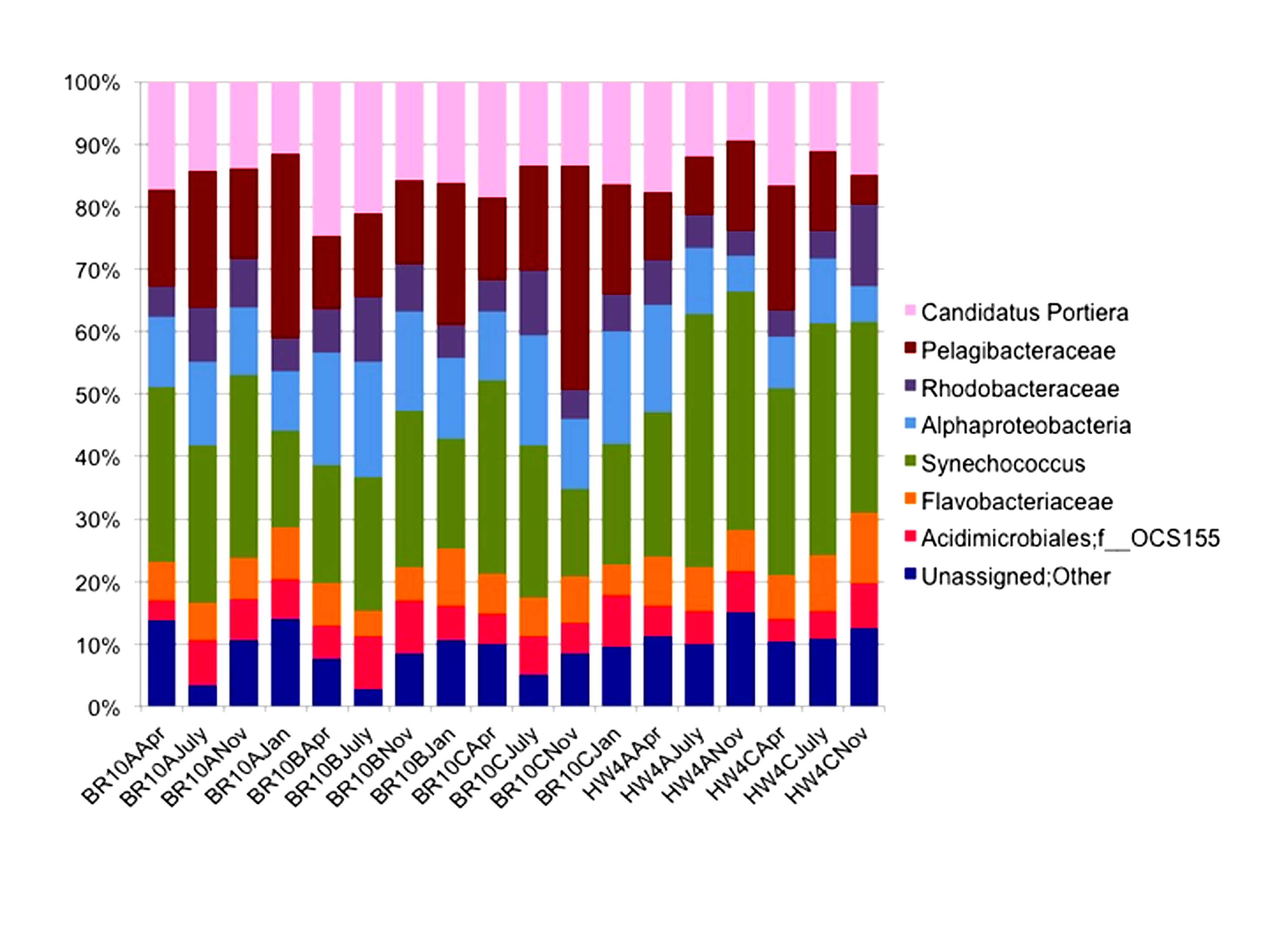

Supplement: Figure S5 — Taxonomic summary of the most abundant taxa (>1%) at the outfall sites. OTUs were determined through QIIME analysis. [file mbo30004-0390-sd5.tif]

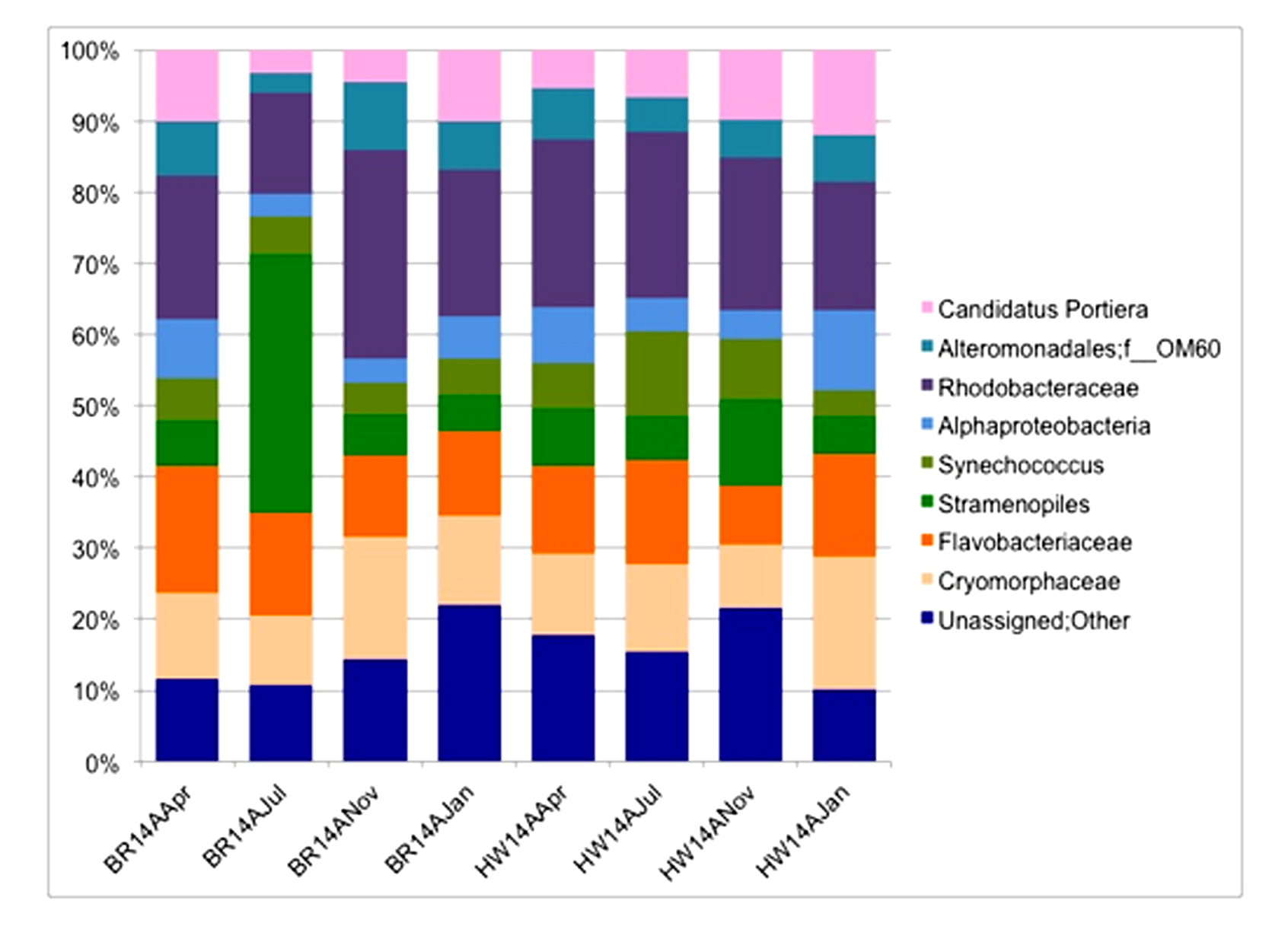

Supplement: Figure S6 — Taxonomic summary of the most abundant taxa (>1%) at the inlet sites. OTUs were determined through QIIME analysis. [file mbo30004-0390-sd6.tif]

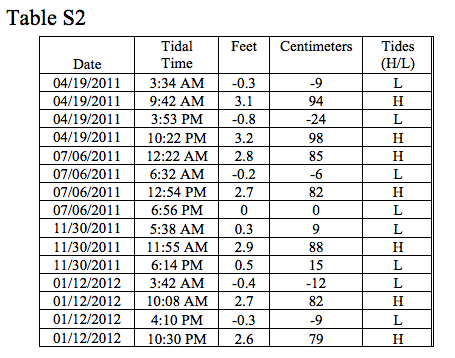

Supplement: Table S2 — Tides and collection times near Fort Lauderdale area outfalls, reefs, and Port Everglades inlet (see Fig. S1). For each date, tides were determined via http://tidesandcurrents.noaa.gov/. [file mbo30004-0390-sd8.tif]

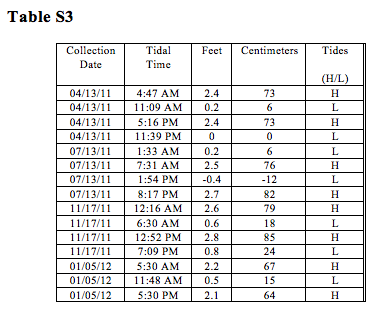

Supplement: Table S3 — Tides and collection times near the Pompano Beach (BR-Broward) area outfalls, reefs, and Hillsboro inlet. For each date, tides were determined via http://tidesandcurrents.noaa.gov/. [file mbo30004-0390-sd9.tif]

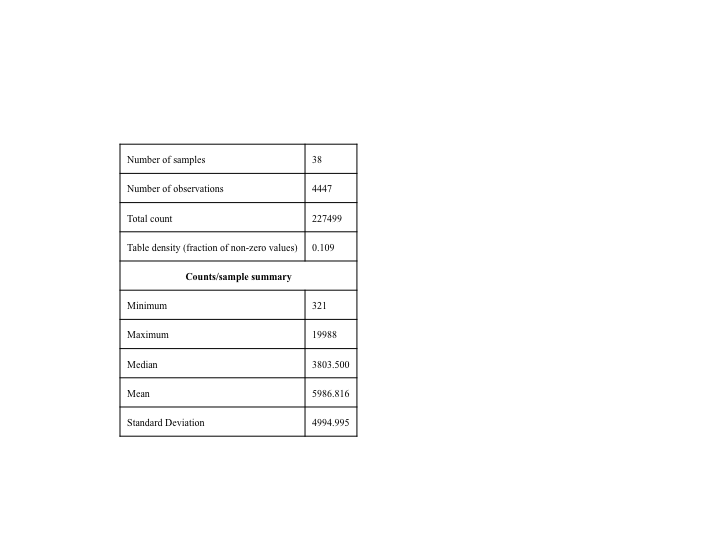

Supplement: Table S4 — Operational taxonomic unit (OTU) summary table. Data were obtained post denoising and post chimera checking. The number of observations is the total number of unique OTUs across all samples. The counts refer to the number of total OTUs found in each sample, and the table density refers to a fraction of non-zero values (McDonald et al. 2012b). [file mbo30004-0390-sd10.tiff]
